# Influence of Zeolite on fatty acid composition and egg quality in Tunisian Laying Hens

**DOI:** 10.1186/1476-511X-11-71

**Published:** 2012-06-07

**Authors:** Imen Fendri, Lamia Khannous, Zouhir Mallek, Al Ibrahim Traore, Néji Gharsallah, Radhouane Gdoura

**Affiliations:** 1 Unité de recherche Toxicologie - Microbiologie Environnementale et Santé (UR11ES70), Faculté des Sciences de Sfax, Université de Sfax, Sfax, Tunisia; 2 Centre Vétérinaire de Recherche, Sfax, Tunisia; 3 Laboratoire national de santé publique, Ouagadougou, Burkina Faso; 4 Laboratoire des Biotechnologies Végétales Appliquées à l’Amélioration des Cultures, Faculté des Sciences de Sfax, Université de Sfax, Sfax, Tunisia

**Keywords:** Zeolites, Egg production, Eggshell quality, Laying hen, Fatty acids

## Abstract

**Background:**

The health benefits of omega-3 and omega-6 polyunsaturated fatty acids (PUFA) are generally recognized. Unfortunately, in most Mediterranean countries, the recommended daily intake of these compounds is rarely met. Therefore, enrichment of commonly occurring foods can boost intake of these fatty acids. In this regard, eggs are an interesting target, as they form an integral part of the diet.

**Result:**

Zeolite (Clinoptilolites) was added to Laying Hens feed at concentrations 1% or 2% and was evaluated for its effects on performance of the production and on egg quality. The Laying Hens were given access to 110 g of feed mixtures daily that was either a basal diet or a ‘zeolite diet’ (the basal diet supplemented with clinoptilolite at a level of 1% or 2%). It was found that zeolite treatment had a positive and significatif (p < 0.05) effect on some parameters that were measured like egg height and eggshell strength. While dietary zeolite supplementation tended to/or has no significant effects on total egg, eggshell, yolk and albumen weights. It was found also that zeolite mainly increases level of polyunsaturated fatty acids in egg.

**Conclusion:**

This study showed the significance of using zeolite, as a feed additive for Laying Hens, as part of a comprehensive program to control egg quality and to increase level of polyunsaturated fatty acids on egg.

## Background

Natural zeolites mining which are easier and cheaper than other mines are found in large reserves [1]. It has been known that zeolites are crystalline, hydrated aluminosilicates of alkali and alkaline earth cations, consisting of three-dimensional frameworks of SiSO_4_^4-^and AlO_4_^5-^ tetrahydralinked through the shared oxygen atoms [2,3]. They have the ability to lose and gain water reversibly and to exchange constituent ionic cations without major changes of structure [4-6].

The exploitation of these properties underlies the use of zeolites in wide range of industrial and agricultural applications and particularly in animal nutrition since mid-1960s [2]. Reviews on the chemistry and applications of zeolits in agriculture are given by Mumpton and Fishman [4]. They reported that both ion exchange and absorption properties of natural zeolites can be used to make more efficient use of feed nitrogen in animal nutrition, to reduce intestinal disease in pigs and ruminants and to control the moisture content of animal manure. In this regard, Onogi feeding up to 10% clinoptiolite-tuff in the diet of Leghorm chickens found no significant difference with regard to mortality but found that mass gain feed efficiency were improved on the clinoptiolite diets [7]. He concluded that zeolite should be used to economize on the feed and reduce the moisture content of chicken dropping.

Others studies suggest that dietary zeolites improves weight gain of fatting pigs, feed efficiency and egg production in laying hens [1,8]. These substances were also found to absorb various mycotoxins [2]. In addition, zeolite has been shown to be a beneficial feed additive that exhibits a strong preference for binding nitrogenous cations like NH_4_^+^ and NH_3_ emission [9]. This property has made it in attractive option for a variety of applications in the treatment of livestock and poultry wastes. Zeolite has been investigated as both a livestock, feed additive and a topical manure additive to adsorb NH_3_. Forms of zeolite have been used to remove ammonium from aqueous solutions [10] and help in reducing ammonia production by pullets and laying hens [9] and in broiler houses [11]. It is important to remove ammonia from poultry houses since it has been reported that high atmospheric ammonia in poultry facilities has been linked to damage respiratory tract lining, reduced resistance to respiratory diseases, increased ascites and lower performance [12].

Previous work [13] has shown that Zeolite, when added as feed additive in Tunisian broilers, influences positively meat structure and on the production of omega 3 polyunsaturated fatty acids. It is important to note that food enrichment is probably the best long-term solution to boost intake of long chain n−3 polyunsaturated fatty acid (PUFA) [14]. An interesting route is n−3 PUFA enrichment of eggs through dietary supplementation of laying hens. The laying hen provides a model for conducting large-scale dietary intervention studies with statistically significant numbers of animals [15].

Omega-3 and omega-6 fatty acids are polyunsaturated fats that are essential fatty acids because they cannot be synthesized by the body and must be obtained from the diet [15]. Omega 3 fatty acids have been shown to reduce inflammation and help prevent risk factors associated with chronic diseases such as heart disease, cancer, and arthritis [15]. Omega 3 fatty acids also appear to be important for proper brain and behavioral functions [15]. Epidemiologic studies indicate that populations that consume high amounts of Omega 3fatty acids have lower incidences of breast, prostate, and colon cancers than do those that consume lower amounts of Omega 3 fatty acids [16-18].

Typically, the amount of saturated or monounsaturated fatty acids in eggs is hardly influenced by the lipids in the feed [19]. In contrast, the PUFA content and profile in the egg can be modified through dietary supplementation.

In this regard, a traditional n−3 PUFA source to be added to hens’ diet is flaxseed, a plant source rich in α-linolenic acid. Alternatively, hens are often fed fish oil, which is rich in long chain n−3 PUFA eicosapentaenoic acid (EPA) and docosahexaenoic acid (DHA). A more recent trend is feed supplementation with microalgae as a source of EPA and/or DHA. As previously reported, Mallek et al. showed a positive effect of zeolite on PUFA production in broilers [13].

The objective of this study is so to examine the influence of natural zeolites, givens as supplement to laying hens feed on the fatty acid composition and on characteristics of eggs.

## Materials and methods

### Zeolite composition

Turkish Manisa Gödes zeolite, obtained from Rota Minnig Corporation (Istanbul, Turkey), was used in the present work. Clinoptiolite was the predominant mineral (84%) present in the natural zeolite used as an active substance, at a moisture level of max. 6%. It also contains 65% of SiO_2_, 12% of Al_2_O3, 2.3% of Fe_2_O3 and 2.7% of CaO (Table 1). Particle size varies in a range of 0.2 – 0.5 mm.

**Table 1:** Semi-quantitative mineralogical composition and chemical microanalysis of the natural zeolite, which was added in the diet

	
**Chemical Component**	**%WT**
SiO2	65-71.3
Al2O3	11.5-13.1
Ca O	2.7-5.2
K2O	2.2-3.4
Fe2O3	0.7-1.9
Mg O	0.6-1.2
Na2O	0.2-1.3
TiO2	0.1-0.3
Mineralogical Component	% WT
Clinoptilolite	84
Cristobalite	8
Feldspar	4
Illite	4
Quartz	Traces
Carbonated minerals	< 0.5

### Preparing of foods

The basal food used in the study is standard powdered containing corn and soybean added with mineral vitamin complement (MVC) (Table 2). The food was obtained from SOMEPA factory (Tunisia). Different rations were prepared by adding zeolite at a weight of 0% (control), 1% and 2% food, respectively.

**Table 2:** Composition of feed mixtures (%) added with mineral vitamin complement (MVC) Nutritional values of diet

**Nutrients**	**%**
Protein	18
Cellulose	3,3
Fat	2,9
Energy (Kcal/kg)	2800
Met	0,4
Lys	0,8
Cys	0,73
Ca	3,5
P	0,55

### Experimental step

A total of 90/60 week-old Babcock B-300 layers hens were used in this study. They were put at random into 3 treatment groups (30 laying hens/group). The first group was used as a control group and had provided a feed without zeolite. The second and the third groups had provided with 1 and 2% of zeolite respectively. Layers hens were placed in wire cages (23x50x42 cm). Experiment was conducted in a three-tier cage system with 3 birds in each cage. They were placed in a deep pit house, ventilated both naturally and mechanically, and illuminated both artificially and naturally through windows. The Laying Hens were given access to 110 g of feed mixtures daily. The experimental measure for each group began after 15 days. 30 eggs were taken at random to the laboratory for the determination of egg quality. The animal protocols were approved by the Animal Research Panel of the Committee on Research Practice of the Sidi Thabet Veterinary School in Tunisia.

### Determination of egg quality

The determination of egg quality was carried out by measuring egg length, shell breaking strength, weights of egg, shell, yolk and albumen. All measurements are duplicated and values averages are given.

#### Determination of egg length

The length of each egg was measured to the nearest 0.01 mm with a Cal electronic digital caliper (Mode 6″ Digital Caliper Electronic).

#### Determination of shell breaking strength

The shell breaking strength was measured using a texturometer (Texture Analyser, TA Plus, LLOYD Instruments, England) as reported methods described by Hidalgo et al. [20].

#### Determination of percentage shell

The shell percentage was determined using the following formula:

(1) $$Shell\;\left(\%\right)=\left(\text{Shell weight}\;\left(\text{g}\right)\right)/\left(\text{Egg weight}\;\left(\text{g}\right)\times 100\right)$$

Weight measurement was carried out using a professional scale with precision 0.001 g (Sartorius BP 110 S).

#### Determination of yolk/albumen ratio

Collected eggs were broken to determine albumen weight and yolk weight. Ratio yolk/albumen was determined using following formula:

(2) $$Ratio\;yolk/albumen=\frac{\text{yolk weight}}{\text{Albumen weight}}$$

### Fatty acid composition

Total lipids for fatty acid analysis were extracted from 5-g aliquot samples of thigh according to Folch et al. [21-24]. Fatty acid methyl esters (FAME) were prepared according to Wang et al. [25], and measured by gas chromatography of FAME in a Chrompack CP 900 apparatus fitted with a flame ionization detector. Analytical gas chromatography was carried out on a Hewlett–Packard 6890 gas chromatograph series II (Agilent Technologies, Palo Alto, California, USA) equipped with HP Innowax (30 m x 0.25 mm, 0.25 ml film thickness) capillary column. Each sample was injected with a split ratio of 1:100 and a continuous flow rate of 1.5 ml/min of chromatographic grade helium was used. The oven temperature was initially held for 20 min at 165 °C, ramped at 5 °C/min up to 240 °C and held isothermal for 25 min. Injector and FID detector temperature were held at 250 °C. FAMEs were identified by comparison of their retention time with respect to pure standard purchased from Sigma and analyzed under the same conditions. FAMEs were quantified according to their percentage area, obtained by integration of the peaks. The results were expressed as a percentage of individual fatty acids in the lipid fraction as described by Pordomingo et al. [26].

Main individual fatty acids were grouped in saturated fatty acids (SFA = myristic (C14:0) + palmitic (C16:0) + stearic (C18:0)), monounsaturated fatty acids (MUFA = myristoleic (C14:1) + palmitoleic (C16:1) + oleic (C18:1)) and polyunsaturated fatty acids (PUFA = n-3 + n-6 fatty acids), n-3 fatty acids (linolenic (C18:3) + eicosapentaenoic (EPA; C20:5) + docosapentaenoic (C22:5; DPA) + docosahexaenoic (DHA; C22:6), n-6 fatty acids (linoleic (C18:2) + di-homo-gamma-linolenic (DGLA; C20:3) + arachidonic (AA; C20:4) + docosatetraenoic (adrenic; C22:4). In this study, only SFA, palmitoleic and oleic MUFA and linoleic and linolenic PUFA (among Omega 6 and Omega 3 groups respectively) were analyzed.

## Results and discussion

### Determination of egg quality at experimental step

The quality of egg produced from different laying hens groups was realized measuring the variation of egg length, variation of percentage shell, variation of breaking shell strength, yolk and albumen weights and the ratio yolk/albumen.

#### Variation of egg length

Variation of egg length was determined for the different rations tested (control laying hens and those that were fed on 1% and 2% of zeolite). Determination of egg length was carried out and results represented in Table 3 show a significant increase of egg length mainly when 1% of zeolite was added. In this regard, others studies report the effect of this mineral, added on animal food, on egg production [27].

**Table 3:** Effect of zeolite addition on egg height, eggshell weight and eggshell strength

	**Control**	**1% Zeolite**	**2% Zeolite**
Egg height (cm)	6.052 ± 0.248	6.605 ± 0.838 ^bd^	6.216 ± 0.247 ^b^
Eggshell weight (g)	6.65 ± 0.941	6.73 ± 0.823	6.31 ± 0.718
Eggshell Strength (N/m)	32.96 ± 0.29	37.01 ± 0.55 ^c^	38.6 ± 0.54 ^c^

Each value is the mean of three determinations followed by standard deviation.

^a^ Significant differences between the control/1% Zeolite and control/2% Zeolite p < 0.05.

^b^ Significant differences between the control/1% Zeolite and control/2% Zeolite p < 0.01.

^c^ Significant differences between the control/1% Zeolite and control/2% Zeolite p < 0.001.

^d^ Significant differences between 1% Zeolite/2% Zeolite p < 0.05.

#### Effect of zeolite on eggshell weight and shell strength

The result of variation of shell strength for the different rations tested was represented in Table 3. The shell strength was significantly increased (p < 0.001) after of addition zeolite in feed laying hens. These results are in agreement with the finding of Olver [28]. Authors reported, since 1989, that adding zeolite to the diet of poultry improves the process of egg shells strength without deleterious effects on the contents of the egg itself [28].

#### Variation of egg weight, yolk and albumen weights

Table 4 shows the variation of egg weight, yolk and albumen weights measured at different rations tested in this work. A slight increase of egg weight was observed when 1% of zeolite was added. Results show also a positive effect of zeolite on albumen weight. 1% seems to be the best concentration of zeolite added in feed which increase albumen mass to about 2% comparing with control albumen mass. However, results indicate that zeolite has no significant effect on yolk mass when 1% of zeolite was added and a slight increase of yolk mass (p < 0.05) after addition of 2% of zeolite. Kermanshahi et al. [29] reported in previous work that natural zeolite has no significant effect effect on mass’s yolk or mass’s albumen but has an effect on yolk color [29]. Finally, our study shows that dietary zeolite has no significant effect on ratio Yolk/albumen comparing with the control (Table 4). These results agree with previous report described by Öztürk et al. [30].

**Table 4:** Effect of zeolite addition on egg, yolk and albumen weights and on the ratio yolk/albumen

	**Control**	**1% Zeolite**	**2% Zeolite**
Total egg weight (g)	63.62 ± 5.965	68.18 ± 6.983 ^a^	66.83 ± 5.561
Egg yolk weight (g)	22.82 ± 2.595	23.4 ± 2.92	24.5 ± 2.585 ^a^
Egg albumen weight (g)	34.15 ± 4.341	38.04 ± 4.724 ^b^	36.02 ± 3.85
Ratio Yolk/albumen	0.68 ± 0.11	0.62 ± 0.08 ^d^	0.69 ± 0.08 ^d^

Each value is the mean of three determinations followed by standard deviation.

^a^ Significant differences between the control/1% Zeolite and control/2% Zeolite p < 0.05.

^b^ Significant differences between the control/1% Zeolite and control/2% Zeolite p < 0.01.

^c^ Significant differences between the control/1% Zeolite and control/2% Zeolite p < 0.001.

^d^ Significant differences between 1% Zeolite/2% Zeolite p < 0.05.

### Yolk fatty acid composition

It is essential to note that from the standpoint of vascular disease prevention, n-3 PUFAs are the most important and extensively studied class of essential PUFA. n-3 and n-6 PUFAs are termed “essential” FA and must be obtained from the diet because humans lack the Δ12- and Δ15-desaturases necessary to insert a double bond at the n-3 or n-6 position of an FA carbon chain. The difference between the two essential PUFA is based on the location of the first double bond of the molecule counting from the methyl end of the FA [26].

As previously reported, Mallek et al. showed a positive effect of zeolite on PUFA production in broilers [13]. In the present experiment, fatty acids content was determined in egg yolks collected from control and zeolite-fed hens. Addition of a natural mineral, the zeolite, was shown to increase significantly (p<0.05) the level of total n−3 fatty acids in eggs fat (Table 5), with this response being primarily related to higher levels of linolenic acid. Similar results were also reported by Lawlor et al. [31] using fed diets containing microencapsulated fish oil. Authors showed that DHA and EPA transfer significantly (p<0.05) to table eggs.

**Table 5:** Effect of Zeolite dietary supplementation on fatty acid composition (% total fatty acids).

**Fatty acids (%)**	**Control**	**1% Zeolite**	**2% Zeolite**
Saturated fatty acids (SFA)			
Myristic acid (C14:0)	0.355 ± 0.005	0.33 ± 0.00 ^c^	0.325 ± 0.005 ^c^
Palmitic acid (C16:0)	26.465 ± 0.255	25.925 ± 1.505	24.6 ± 0.06
Stearic acid (C18:0)	9.04 ± 0.33	9.025 ± 0.785	7.315 ± 0.825 ^ad^
Mono-unsaturated fatty acids (MUFA)			
Palmitoleic acid (C16:1)	3.265 ± 0.175	3.5 ± 0.1	3.925 ± 0.285 ^b^
Oleic acid (C18:1)	45.565 ± 0.005	46.79 ± 0.35 ^a^	47.54 ± 0.76 ^b^
Poly-unsaturated fatty acids (PUFA)			
Linoleic acid (C18:2)	13.06 ± 0.11	13.29 ± 1.08 ^d^	15.56 ± 0.725 ^bd^
Linolenic acid (C18:3)	0.25 ± 0.03	0.275 ± 0.035 ^d^	0.465 ± 0.165 ^ad^

Each value is the mean of three determinations followed by standard deviation.

^a^ Significant differences between the control/1% Zeolite and control/2% Zeolite p < 0.05.

^b^ Significant differences between the control/1% Zeolite and control/2% Zeolite p < 0.01.

^c^ Significant differences between the control/1% Zeolite and control/2% Zeolite p < 0.001.

^d^ Significant differences between 1% Zeolite/2% Zeolite p < 0.05.

It is important to note that PUFAs (linoleic acid and alpha-linolenic acid) have displayed protection against lipid peroxidation increasing the levels of several cellular antioxidants such as ascorbic acid, a-tocopherol and GSH [32]. In the present work, linoleic acid increased significantly (p<0.01) compared to the control mainly when 2% of zeolite was added.

The effects of zeolite on two monounsaturated fatty acids (MUFA) were showed in Table 5. When 1% of Zeolite was added, the percentage of 18:1 oleic acid increased compared with control. However there is no significant increase of 16:1 palmitoleic acid percentage, again likely as a result of a reduction in Δ9 desaturase activity. Similar results were reported by Cachaldora et al. [33]. Authors showed that inclusion of 50 g lard/kg only increased slightly the monounsaturated FA content (by 3.3%) and had little effect on the rest of the FA profile with respect to the control diets with no fat added.

Addition of Zeolite reduced clearly the level of Myristic (C14:0) saturated fatty acid (p<0.001) in yolk eggs (Table 5) and this was attributed to a slight reduction in C18:0 stearic acid when 2% of zeolite was added. No significant difference in C16:0 palmitic acid content was observed compared with control. Fraeye et al. reported that typically, the amount of saturated or monounsaturated fatty acids in eggs is hardly influenced by the lipids in the feed [19]. In contrast, the PUFA content and profile in the egg can be modified through dietary supplementation.

Finally, it is important to note that n−3 PUFA are susceptible to oxidation. Therefore, increasing the level of these sensitive fatty acids in egg yolk may bring about a higher extent of lipid oxidation, which could impair sensorial quality. Furthermore, lipid oxidation products can damage biological tissues and have been related to several diseases such as cancer and atherosclerosis [34]. We did not observe any differences in trans-fatty acids when zeolite was added compared to control eggs (data not shown) indicating that no oxidative damage in egg yolks was occurred during treatment.

## Conclusion

Plant and animal fats contain relatively minor levels of n-3 fatty acids, with the exception of those plant oils that are rich in ALA, such as flax and canola. In order to achieve products, such as plant oils or animal fats, which contain longer chain n-3 PUFA, additional approaches are necessary [31]. One example of such an approach is the n-3 enriched egg.

Significant dietary effects of feeding zeolite are observed for egg height and eggshell strength at the recording period, while dietary zeolite supplementation tended to/or has no significant effects on total egg, eggshell, yolk and albumen weights. When zeolite is added, the composition of egg yolk fatty acids content is significantly modified. The present experiment shows the significance of using zeolite, as a feed additive for Laying Hens, as part of a comprehensive program to control egg quality and to increase level of polyunsaturated fatty acids on egg.

## Competing interests

The authors declare that they have no competing interests.

## Authors’ contributions

IF, LK, ZM and, AT designed the experiments, analyzed the data and drafted the manuscript. NG and RG conceived research and approaches and have given final approval of the manuscript to be published. All authors read and approve the final manuscript.
